# Evaluation of Orthostatic Hypotension in Patients With Idiopathic Parkinson’s Disease Utilizing the Head-Up Tilt Test

**DOI:** 10.7759/cureus.104363

**Published:** 2026-02-27

**Authors:** B B V Ramanan, Suresh C Thirunavukarasu, Devi Varadharaj, Durai Arumugam, Jayasri Poyyadhappan, Nivethini Natarajan, Sigilipalli Vijaykumar

**Affiliations:** 1 General Medicine, Swamy Vivekanandha Medical College Hospital and Research Institute, Namakkal, IND; 2 Neurology, Indira Gandhi Government General Hospital and Post Graduate Institute, Puducherry, IND

**Keywords:** falls, head-up tilt test, modified hoehn and yahr scale, orthostatic hypotension, parkinson's disease

## Abstract

Introduction: Parkinson’s disease (PD) is a neurodegenerative disorder, affecting individuals aged 60 and older, with rapidly increasing prevalence in aging populations, particularly in India. Apart from classical motor symptoms, nonmotor symptoms, particularly orthostatic hypotension (OH), significantly affect the quality of life, increasing the fall risk that contributes to increasing morbidity and mortality. This single-center, prospective, cross-sectional observational study evaluates OH, a key cardiovascular autonomic dysfunction, in idiopathic PD patients using the head-up tilt test (HUTT) to assess its prevalence and correlation with disease duration and severity. Conducted from July 2023 to June 2024 at the Department of Neurology, Indira Gandhi Government General Hospital and Post Graduate Institute, Puducherry, India, the study enrolled 114 patients diagnosed according to UK Brain Bank criteria, excluding confounders such as diabetic neuropathy or cardiovascular comorbidities.

Methods: Patients underwent detailed clinical evaluation, demographics, PD duration, autonomic symptom history, and Modified Hoehn and Yahr staging, and the HUTT was commenced at 60° after 30 minutes of supine rest. Systolic blood pressure (SBP), diastolic blood pressure (DBP), mean arterial pressure, and heart rate were obtained every five minutes before tilt; one, three, and five minutes during tilt; and one minute after tilt, as indicated for patient safety. OH was defined as a drop in SBP ≥20 mmHg and/or DBP ≥10 mmHg within three to five minutes. Data analysis used Statistical Package for the Social Sciences version 20 (IBM Corp., Armonk, NY) with chi-square test, analysis of variance, t tests, and Pearson correlation (p < 0.05 significant). Ethical approval was obtained from the institution’s ethics committee, and informed consent was obtained to ensure confidentiality.

Results: The mean age was 65 ± 11.19 years, 62.28% were male, and the mean disease duration was 2.75 ± 1.93 years. OH prevalence was 28% (32/114 patients). No gender association was observed (p = 0.180), but strong links with duration (p < 0.00001; 0% in less than one year, 100% in greater than five years) and H&Y stage (p < 0.00001; 7.4% stage 1, 100% stage 3) were noticed. Disease duration positively correlated with H&Y stage (r = 0.459, p = 0.0001).

Discussion: OH increases progressively with PD duration and motor severity, reflecting autonomic neurodegeneration independent of dopaminergic loss, consistent with prior studies. The HUTT’s sensitivity detected asymptomatic cases, which are vital for fall mitigation. HUTT proved superior to static measures for early detection. Limitations of the study include a single-center design and the absence of a control group. Routine screening via HUTT is recommended for advanced PD to prevent falls/syncope via interventions (e.g., hydration and midodrine).

Conclusion: OH affects 28% of idiopathic PD patients, strongly tied to duration and H&Y stage; HUTT enables proactive management to enhance the quality of life.

## Introduction

Parkinson’s disease (PD) is the second most prevalent neurodegenerative disorder among the elderly after Alzheimer’s disease [1]. It affects a growing number of patients globally and in India, escalating the associated health burden [2]. Beyond the hallmark motor features such as tremor, rigidity, bradykinesia, and postural instability, nonmotor symptoms have garnered increasing focus in PD. These include olfactory loss, sleep disturbances, apathy, anxiety, depression, cognitive decline, and autonomic dysfunction. Autonomic issues, in particular, stand out as key manifestations that profoundly impair patients' quality of life, imposing substantial challenges on individuals, their caregivers, and healthcare economics [3,4]. In PD, autonomic dysfunction can affect multiple components of the autonomic nervous system (ANS), including the sympathetic noradrenergic system (SNS), sympathetic cholinergic system, sympathetic adrenomedullary system (SAS), parasympathetic nervous system (PNS), and enteric nervous system [1]. Clinically, this results in a wide range of symptoms and signs involving the cardiovascular, gastrointestinal, urinary, reproductive, integumentary, and other systems. The cardiovascular system is mainly dominated by SNS, SAS, and PNS [1]. Autonomic dysfunction in PD likely stems from lesions in key central sites, including the hypothalamus, basal ganglia, reticular formation, locus coeruleus, and dorsal motor nucleus of the vagus. In addition to these preganglionic regions, postganglionic sympathetic neurons and other peripheral autonomic elements are also involved [5]. The common cardiovascular autonomic dysfunction in PD is orthostatic hypotension (OH), which clinically manifests as dizziness, postural instability, vertigo, blurred vision, syncope, and fatigue [6]. OH is recognized as a modifiable risk factor for cognitive impairment in PD [1]. Additionally, OH in PD has been linked to postural instability and increased risk of falls. The resulting morbidity from falls may necessitate active management. In the initial phase of PD, patients may remain asymptomatic, yet subclinical cardiovascular dysautonomia often lurks beneath the surface. Early detection of these causes enables clinicians to tailor therapies proactively, mitigating dysautonomia-related falls alongside motor symptoms [7]. Most earlier studies assessed cardiovascular autonomic control using morphological and functional approaches. The limitations of these studies were that they did not dynamically evaluate the ANS. This study aimed to assess OH in PD patients via the head-up tilt test (HUTT). HUTT serves as a proactive maneuver to detect an individual's vulnerability to blood pressure (BP) drops [8]. We analyzed the spectral characteristics of BP responses to orthostatic stress induced by HUTT in these patients.

## Materials and methods

Study design and setting

This was a single-center, prospective, cross-sectional observational study in which participants were enrolled and underwent a single standardized assessment without follow-up at the Department of Neurology, Indira Gandhi Government General Hospital and Post Graduate Institute (IGGGH&PGI), Puducherry. The study population was inpatients and outpatients diagnosed with idiopathic PD in the medical and neurology wards of IGGGH&PGI, Puducherry, between July 2023 and June 2024.

Inclusion criteria

All cases of idiopathic PD diagnosed according to UK Brain Bank criteria were included.

Exclusion criteria

Other causes of Parkinsonism, history of diabetic neuropathy, history of other peripheral/autonomic neuropathies, patients with cardiovascular, respiratory, and gastrointestinal diseases that could interfere with cardiac autonomic control, any abnormalities on routine chest radiography or electrocardiography, abnormal liver and renal function tests, patients on medications that affect cardiovascular autonomic function, excluding anti-Parkinsonian drugs, history of smoking and alcohol, patients with active infections, and patients with mental illness were excluded.

Ethical clearance

Ethical clearance was obtained from the Institutional Ethical Committee before the start of the study (GHIEC/2023/13).

Methodology

Patients with idiopathic PD who consented to participate in the study were evaluated with a detailed history, including age, sex, disease duration, clinical features of autonomic neuropathy, and treatment history. Assessment of the severity of the disease was done by the Modified Hoehn and Yahr staging. All relevant blood and radiological test reports were included in the documentation. The study participants were asked to report to the neurology department for recording the ECG and HUTT. Head-up tilt table testing was typically performed in the neurophysiology laboratory using a special motorized tilt table that rises to 90° above the supine position. The test duration was approximately 20 minutes. This study employed a hydraulically driven head-up tilt table featuring footboard support (7’ × 3’). Calibrated for upright tilts ranging from 60° to 90°, it incorporated sensors for precise angle detection, ensuring a smooth transition from the supine position. Participants were gently secured by straps to avoid falls. Continuous electrocardiographic and noninvasive BP monitoring leads were connected to each subject. Following 30 minutes of supine rest, the HUTT commenced. Systolic blood pressure, diastolic blood pressure, mean arterial pressure, and heart rate were obtained every five minutes before; one, three, and five minutes during tilt; and one minute after tilt, as indicated for patient safety. Drugs like antihypertensives, diuretics, and caffeine were withheld 48 hours before testing, and the test was done in the morning hours between 11 AM and 2 PM. OH was defined as a sustained fall in systolic BP ≥20 mmHg and/or diastolic BP ≥10 mmHg from supine baseline, occurring between three and five minutes of head-up tilt. For statistical evaluation, the nadir values recorded between three and five minutes after tilt were selected. An emergency tray was kept ready to address any adverse events arising from upright tilting of participants.

Statistical analysis

Statistical analyses were performed using Statistical Package for the Social Sciences (version 20; IBM Corp., Armonk, NY). Continuous variables were reported as mean ± standard deviation (SD) or median (interquartile range), as appropriate, after assessing normality. Categorical variables were presented as frequencies and percentages. The prevalence of OH was calculated with corresponding 95% confidence intervals (CIs) using binomial estimation. Group comparisons were performed using the chi-square test for categorical variables and the Student t-test or analysis of variance for continuous variables, as appropriate. Correlation between disease duration and Modified Hoehn and Yahr (H&Y) staging was assessed using Pearson's correlation coefficient. A two-tailed p value of <0.05 was considered statistically significant. Subgroup analyses were exploratory, and no formal adjustment for multiple comparisons was applied.

Limitations

The limitations include the following: this is a single-center study, the study lacked an age- and sex-matched healthy control group, and patients with Modified H&Y staging 4 and 5 were not represented in the study, likely due to limited mobility or poor general condition.

## Results

Between July 2023 and June 2024, 114 patients were included in the study. The mean age was 65 ± 11.19. The majority of patients (84.21%) were aged 51-80 years, with the peak incidence in the 51-60-year age group (28.95%). Among 114 patients, 71 (62.28%) were male and 43 (37.72%) were female patients, with a male:female ratio of 1.65:1; 42.98% were from the rural population, and 57.02% were from the urban population; 23.68% patients had disease duration of less than one year, 34.21% of patients had disease duration between one and two years, 9.65% of patients had disease duration between two and three years, 10.53% patients had disease duration between three and four years, 13.16% patients had disease duration between four and five years, and only 8.77% patients had disease duration beyond five years. Twenty-seven patients had modified H&Y staging 1, 41 patients had modified H&Y staging 1.5, 21 patients had modified H&Y staging 2, 20 patients had modified H&Y staging 2.5, five patients had modified H&Y staging 3, and no patients were in modified H&Y staging 4 or 5. The prevalence of OH was 28% (95% CI: 19.8-36.2). There was no statistically significant association (p = 0.180) between gender and the occurrence of OH in idiopathic PD. There is a statistically strong association (p < 0.00001) between the duration of PD and the occurrence of OH, that is, the prevalence of OH increases significantly with longer disease duration, suggesting progressive autonomic dysfunction over time. In this study, two out of 27 patients in stage 1 had OH, seven out of 41 patients in stage 1.5 had OH, seven out of 21 in stage 2 had OH, 11 out of 20 patients in stage 2.5 had OH, and five out of five patients in stage 3 had OH. The p value is highly significant (p < 0.00001), indicating a strong association between disease severity (as per Modified H&Y staging) and the occurrence of OH. The frequency of OH increased progressively with advancing H&Y stage, with 100% of patients in stage 3 exhibiting OH. This supports the finding that autonomic dysfunction, particularly cardiovascular, becomes more prevalent with increasing motor severity in PD. Also, there is a positive correlation between disease duration and modified H&Y staging, with an r value of 0.45 and a significant p value of <0.0001.

## Discussion

**Figure 1:**
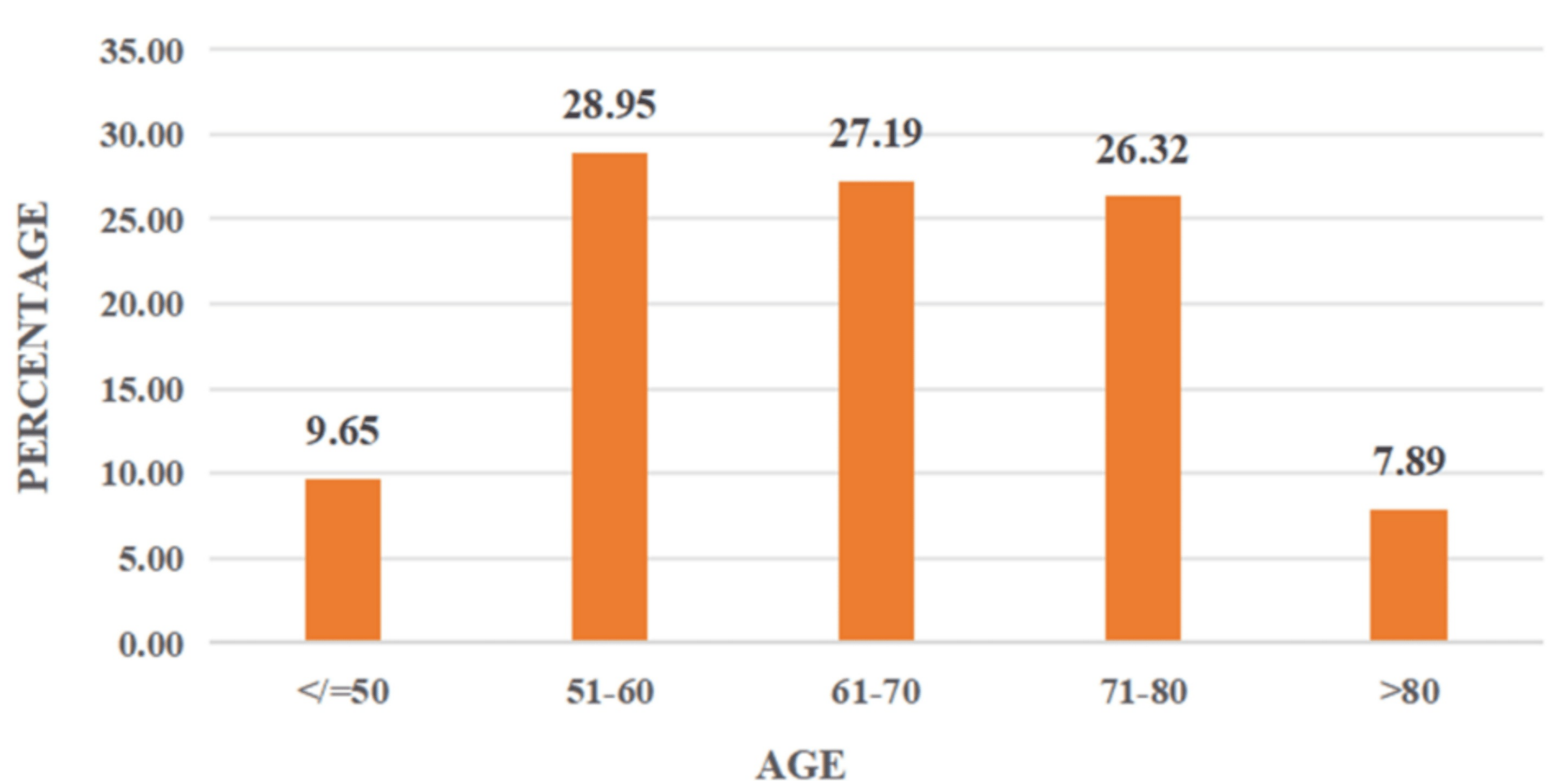
Age distribution

In our study of 114 patients, the age range was between 40 and 85, with a mean age of 65 ± 11.192. The incidence of OH was maximum between 51 and 60 years of age, which comprises 28.95% (33 cases). The relatively low percentage of patients under 50 years of age (9.65%) reflects the rarity of young-onset PD, which typically comprises about 5%-10% of all PD cases [9]. The relatively low representation of patients beyond 80 years (7.89%) could be due to multiple factors, including increased frailty and coexisting comorbidities, which may limit participation in structured clinical testing like HUTT. A study by Kim et al. [10] showed that the mean age (±SD) was 68.4 ± 10.4 years, and a study by Yalcin et al. [11] showed that the mean age (±SD) was 73 ± 7.9 years. The age distribution of the study population is illustrated in Figure 1.

**Figure 2:**
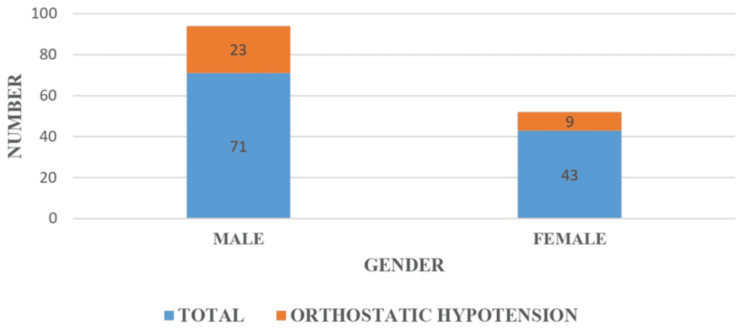
Comparison between gender and orthostatic hypotension

In the present study, 71 (62.28%) were male and 43 (37.72%) were female patients. This male predominance is consistent with global and regional epidemiological data on PD, which indicate higher incidence and prevalence in male than in female patients. Biological factors, such as the neuroprotective effects of estrogen, may play a role in delaying the onset or reducing the severity of PD in female patients. Experimental models have shown that estrogen may influence dopaminergic activity and oxidative stress in the substantia nigra, potentially offering some protection against neurodegeneration. Additionally, lifestyle factors, occupational exposures (e.g., to pesticides or heavy metals), and differences in healthcare-seeking behavior may contribute to the observed gender gap [12]. Despite the higher number of male participants in our cohort, the prevalence of OH did not show a statistically significant gender-based difference. Among the 32 patients who developed OH, 23 (71.88%) were male and nine (28.12%) were female patients. Although the absolute number was higher in male patients, this proportion closely mirrors the overall gender distribution in the sample. This suggests that while idiopathic PD may be more prevalent in male patients, the development of autonomic dysfunction, specifically OH, may not be strongly influenced by gender alone. A study by Valente et al. showed a male preponderance (84%) [13]. A study by Yalcin et al. [11] reported female predominance (58.8%) in PD but found no significant gender-related difference in the autonomic dysfunction profile. Further studies with larger cohorts and controlled confounding factors are needed to clarify these trends. Clinicians should, therefore, harbor a high index of suspicion for autonomic dysfunction in PD patients of both sexes, particularly in early stages where symptoms may be subtle or underreported. The association between gender and the occurrence of orthostatic hypotension is shown in Figure 2.

In this study, the duration of idiopathic PD patients ranged from less than one year to over five years, with a mean duration of 2.75 ± 1.93 years. The largest proportion of patients (34.21%) had a disease duration of one to two years, followed by 23.68% with a duration of less than one year. Only 8.77% of patients had been living with the disease for more than five years in our study. This pattern reflects a tendency for earlier disease stages to be more frequently represented in hospital-based studies, possibly because of more proactive symptom monitoring and management in the early years after diagnosis. The lower number of patients in the greater than five-year category may be attributed to decreased mobility, greater disability, or comorbidities that limit hospital access or participation in structured testing such as the HUTT.

**Figure 3:**
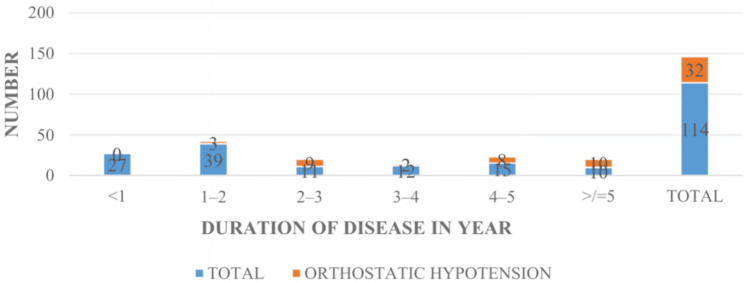
Comparison between duration of disease and orthostatic hypotension

The prevalence of OH showed a clear increasing trend with longer disease duration. Notably, no patients with a disease duration of less than one year exhibited OH. Only 7.69% (95% CI: 0-16) had OH within one to two years of disease duration. This proportion increased dramatically to 81.81% (95% CI: 59-100) in those with two to three years, 16.7% (95% CI: 0-37.8) in those in three to four years, 53.33% (95% CI: 28.1-78.6) in four to five years, and 100% (95% CI: 69.2-100) in those with greater than five years of disease duration (n = 10). These findings suggest a progressive impairment of autonomic function over time in PD. As the disease advances, neurodegeneration may extend to autonomic centers such as the dorsal motor nucleus of the vagus, hypothalamus, and intermediolateral cell column of the spinal cord, leading to cardiovascular dysautonomia and OH. Our results are supported by previous studies, such as Mesec et al., which demonstrated that cardiovascular reflexes progressively deteriorate in PD patients over time [14]. Also, the Yalcin et al. study showed that disease duration in PD patients with OH (82.7 ± 60.7 months) was significantly higher than in PD patients without OH (38.5 ± 46 months) (p < 0.01) [11]. The increasing trend of OH with disease duration underscores the importance of routine screening for autonomic dysfunction in PD patients, particularly as the disease progresses. Early identification allows for timely management strategies, such as lifestyle modifications and pharmacological interventions, to mitigate the risk of falls. These findings also reinforce the relevance of dynamic autonomic testing, such as the HUTT, especially in patients beyond the early disease phase, where symptoms may be subtle or attributed to aging or medication effects. The distribution of patients according to duration of disease is summarized in Table 1, and the relationship between disease duration and the prevalence of orthostatic hypotension is depicted in Figure 3.

**Table 1:** Distribution by duration of disease

Duration of disease (years)	n (%)
<1	27 (23.68)
1-2	39 (34.21)
2-3	11 (9.64)
3-4	12 (10.52)
4-5	15 (13.15)
≥5	10 (8.77)
Total	114 (100)

In this study, the majority were in the early to moderate stages of the disease, as assessed by the modified H&Y scale. The most common stage was 1.5, accounting for 35.97% of patients, followed by stage 1 (23.68%), stage 2 (18.42%), and stage 2.5 (17.54%). Only a small proportion of patients were in stage 3 (4.39%), and none were in stage 4 or 5. This distribution suggests that patients were predominantly in the early to mid-stages of PD, possibly because of better mobility and a willingness to participate in testing protocols such as the HUTT. The absence of late-stage (stages 4 and 5) patients could be attributed to severe disability, comorbidities, or logistical challenges that restrict access to outpatient or neurophysiology laboratory-based assessments.

**Figure 4:**
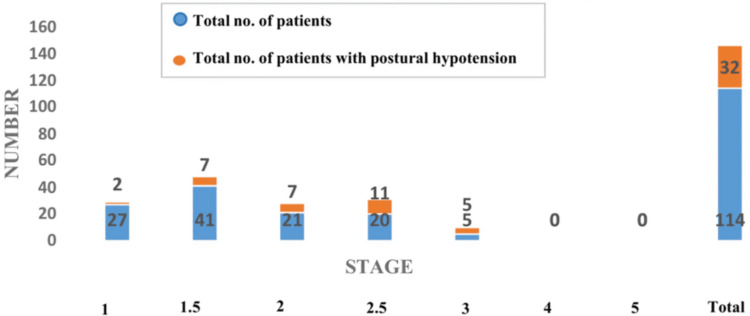
Comparison between modified H&Y staging and orthostatic hypotension H&Y: Hoehn and Yahr

A progressively increasing trend in the prevalence of OH was observed with advancing H&Y stage: only 7.4% (95% CI: 0-17.3) in stage 1 had OH. In stage 1.5, 17.07% (95% CI: 5.6-28.6) had OH. In stage 2, OH was seen in 33.33% (95% CI: 13.2-53.5). A marked increase was observed in stage 2.5, where 55% (95% CI: 33.2-76.8) exhibited OH. Strikingly, all patients (n = 5) in stage 3 had OH (95% CI: 47.8-100). No patients were available for evaluation in stages 4 and 5. In our cohort, among 32 patients who had OH on tilt-table testing, only 13 patients were symptomatic for OH. Among them, the majority were in stage 2.5 (n = 9), while two patients were each in stage 1 and 2. These findings highlight a strong correlation between disease severity and the presence of OH, with a significant increase in prevalence occurring from stage 2.5 onward. The modified H&Y staging reflects the degree of motor impairment and disease progression, and this study supports the notion that autonomic dysfunction escalates with advancing motor disability. The increasing prevalence of OH with higher H&Y stages may be explained by more extensive neurodegeneration affecting both central and peripheral autonomic regulatory centers, including the medullary autonomic centers, intermediolateral columns of the spinal cord, and peripheral sympathetic neurons. As PD progresses, these structures become increasingly impaired, contributing to cardiovascular dysautonomia. Our findings are in line with previous studies. For example, in a study by Kim et al., 106 had mild and unilateral disease (modified H&Y stage score 1 and 1.5), 51 had moderate and bilateral disease (modified H&Y stage score 2 and 2.5), and 31 had severe motor symptoms (modified H&Y stage score ≥3). The proportion of OH in the study is as follows: mild vs. moderate vs. severe = 29.2% vs. 31.4% vs. 29.0%, χ^2^ = 0.085, p = 0.959 [10]. Furthermore, the strong correlation observed between H&Y stage and OH in our study reinforces the importance of routinely evaluating for OH, especially in patients beyond stage 2. Early detection of OH in moderate to advanced PD stages is essential, as it may reduce the risk of falls, syncope, and related morbidity. Interventions like pharmacotherapy, compression stockings, hydration, and medication adjustments can significantly improve patient safety and quality of life. The distribution of patients based on modified H&Y staging is shown in Table 2, and the comparison between modified H&Y staging and orthostatic hypotension is presented in Figure 4.

**Table 2:** Distribution by modified H&Y staging H&Y: Hoehn and Yahr

Modified H&Y staging	n (%)
1	27 (23.68)
1.5	41 (35.96)
2	21 (18.42)
2.5	20 (17.54)
3	5 (4.38)
4	0 (0)
5	0 (0)
Total	114 (100)

**Figure 5:**
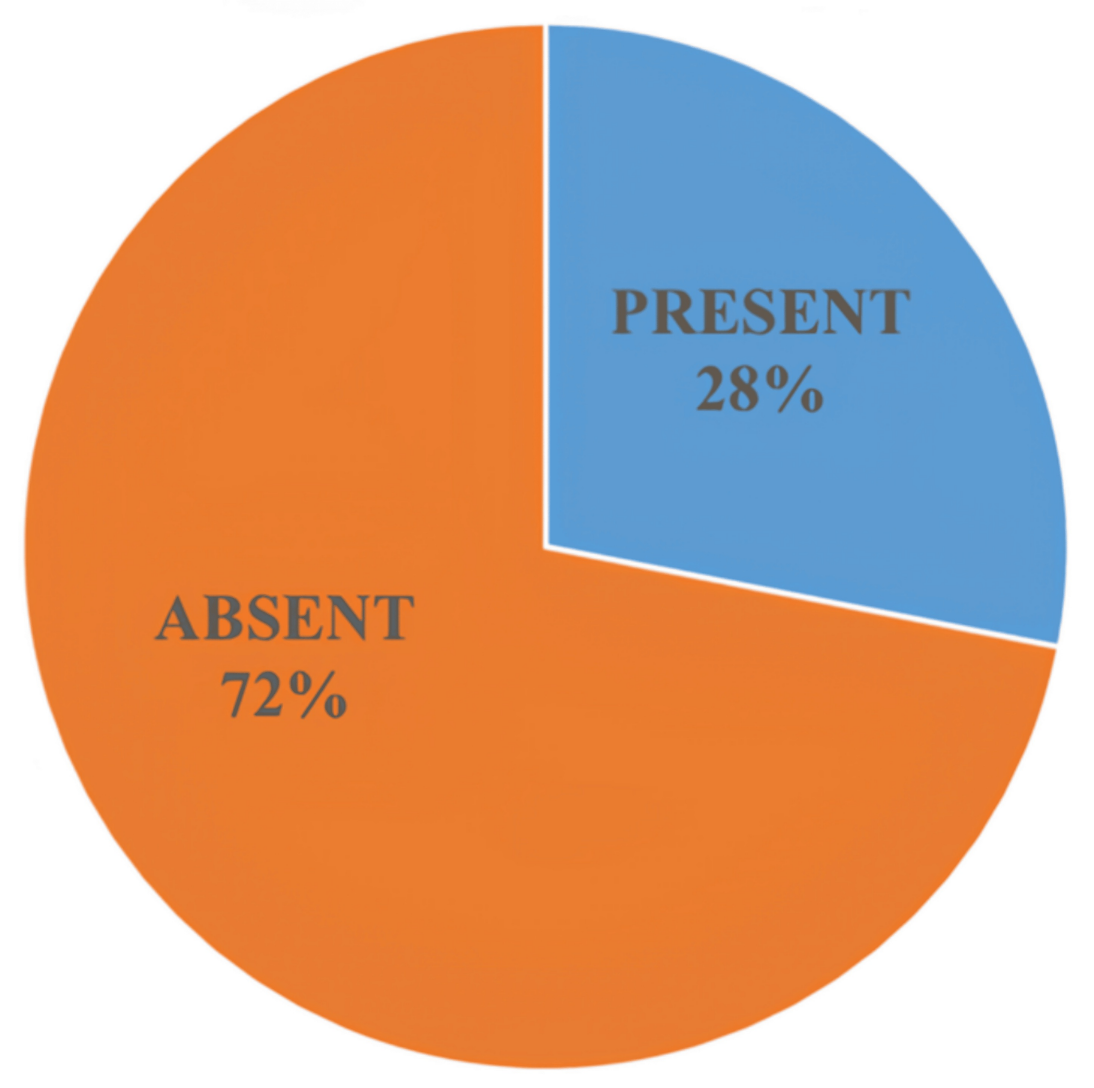
Prevalence of orthostatic hypotension

In the present study, only 28% (95% CI: 19.8-36.2) had OH. This prevalence falls within the lower range reported in the literature, where estimates vary widely from approximately 20% to 50%. The relatively modest prevalence observed in our cohort may be explained by several factors. First, the majority of patients in our study were in early to mid-stages of the disease, with very few patients in advanced stages and none in stages 4 and 5. Second, the mean disease duration in our cohort was relatively short (2.75 ± 1.93 years). Additionally, strict exclusion criteria were applied, including the exclusion of patients with diabetes, cardiovascular disease, other autonomic neuropathies, and other medications affecting autonomic function. While all these strengthened internal validity, they reduced the observed prevalence compared to real-world cohorts. The overall prevalence of orthostatic hypotension in the study cohort is demonstrated in Figure 5.

**Figure 6:**
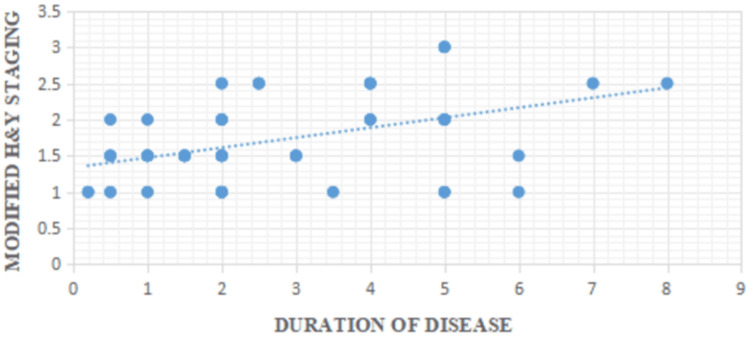
Correlation between duration of disease and modified H&Y staging

In the present study, a statistically significant positive correlation was observed between PD duration and the modified H&Y staging, with an r value of 0.458 and a p value of <0.0001. This indicates that as the duration of the disease increases, the severity of motor symptoms, as measured by H&Y staging, also tends to increase. This finding aligns with the natural history of PD, which is characterized by progressive neurodegeneration. Over time, degeneration of the dopaminergic neurons in the substantia nigra and other brainstem nuclei leads to worsening of motor function. The modified H&Y staging system, which is widely used to quantify motor disability in PD, thus serves as a reliable surrogate marker of disease progression. This correlation has practical clinical implications. Recognizing the relationship between disease duration and functional decline helps clinicians anticipate future disability, plan timely interventions, and counsel patients and caregivers more effectively. Furthermore, it supports the utility of using disease duration as a contextual variable when assessing the risk of complications such as OH, falls, or cognitive decline. Our findings are consistent with earlier studies by Skorvanek et al., where the disease duration increased from H&Y stage 1 to stage 5, and 4.1% of patients had a disease duration from >10 years in H&Y stage 1 to 67.2% in stage 5, which have demonstrated that both motor and autonomic symptoms in PD tend to become more pronounced with longer disease duration [15]. The positive correlation between disease duration and modified H&Y staging is illustrated in Figure 6.

## Conclusions

This study demonstrated a high prevalence of OH among patients with idiopathic PD, particularly in those with longer disease duration and more severe disease staging. The findings highlight the utility of HUTT in early detection of cardiovascular autonomic dysfunction in patients with PD even during asymptomatic period. Timely identification of OH through dynamic testing like HUTT allows for appropriate clinical interventions that may reduce falls and improve quality of life in affected patients. These findings emphasize the need for routine autonomic evaluation as an integral part of PD management, particularly in those with longer disease duration or advancing motor symptoms even when they are asymptomatic.
